# Delta Variant in the COVID-19 Pandemic: A Comparative Study on Clinical Outcomes Based on Vaccination Status

**DOI:** 10.3390/jpm14040358

**Published:** 2024-03-28

**Authors:** Damiana-Maria Vulturar, Liviu-Ștefan Moacă, Maria Adriana Neag, Andrei-Otto Mitre, Teodora-Gabriela Alexescu, Diana Gherman, Iulia Făgărășan, Ioana Maria Chețan, Claudia Diana Gherman, Oana-Elena Melinte, Antigona Carmen Trofor, Doina-Adina Todea

**Affiliations:** 1 Department of Pneumology, “Iuliu Hatieganu” University of Medicine and Pharmacy, 400332 Cluj-Napoca, Romania; vulturar.damianamaria@elearn.umfcluj.ro (D.-M.V.); fagarasan_iulia@elearn.umfcluj.ro (I.F.); chetan.ioana.maria@elearn.umfcluj.ro (I.M.C.); dtodea@umfcluj.ro (D.-A.T.); 2 Department of Pharmacology, Toxicology and Clinical Pharmacology, “Iuliu Hatieganu” University of Medicine and Pharmacy, 400337 Cluj-Napoca, Romania; maria.neag@umfcluj.ro; 3 Department of Pathophysiology, Faculty of Medicine, “Iuliu Hatieganu” University of Medicine and Pharmacy, 400349 Cluj-Napoca, Romania; andrei.otto.mitre@elearn.umfcluj.ro; 4 4th Department Internal Medicine, “Iuliu Hatieganu” University of Medicine and Pharmacy, 400015 Cluj-Napoca, Romania; teodora.alexescu@umfcluj.ro; 5 Department of Radiology, “Iuliu Hatieganu” University of Medicine and Pharmacy, 400347 Cluj-Napoca, Romania; gherman_diana@elearn.umfcluj.ro; 6 Department of Surgery-Practical Abilities,“Iuliu Hatieganu” University of Medicine and Pharmacy, Marinescu Street, No. 23, 400337 Cluj-Napoca, Romania; gherman.claudia@umfcluj.ro; 7 Discipline of Pneumology, III-rd Medical Department, Faculty of Medicine, “Grigore T. Popa” University of Medicine and Pharmacy, 700115 Iasi, Romania; oana-elena.melinte@umfiasi.ro (O.-E.M.); antigona.trofor@umfiasi.ro (A.C.T.)

**Keywords:** SARS-CoV-2, COVID-19, vaccines, Delta variant, severe disease, ICU admission, mortality

## Abstract

Background: As the global battle against the COVID-19 pandemic endures, the spread of the Delta variant has introduced nuanced challenges, prompting a nuanced examination. Materials and Methods: We performed a multilevel logistic regression analysis encompassing 197 patients, comprising 44 vaccinated individuals (V group) and 153 unvaccinated counterparts (UV). These patients, afflicted with the Delta variant of SARS-CoV-2, were hospitalized between October 2021 and February 2022 at the COVID-19 department of a University Centre in Cluj-Napoca, Romania. We compared patient characteristics, CT lung involvement, Padua score, oxygen saturation (O_2_ saturation), ventilation requirements, dynamics of arterial blood gas (ABG) parameters, ICU admission rates, and mortality rates between the two groups. Results: The UV group exhibited a statistically significant (*p* < 0.05) proclivity toward developing a more severe form of infection, marked by elevated rates of lung involvement, oxygen requirement, ICU admission, and mortality. Conclusion: Our findings underscore the substantial efficacy of the vaccine in diminishing the incidence of severe disease, lowering the rates of ICU admissions, and mitigating mortality among hospitalized patients.

## 1. Introduction

In recent years, the global public health landscape has been profoundly affected by the formidable SARS-CoV-2 infection, leading to the widespread COVID-19 pandemic. Throughout this ongoing health crisis, various variants of SARS-CoV-2 have emerged, contributing to a spectrum of disease severity, from mild to severe [1,2]. A pivotal response to combat this pandemic has been the rapid development and deployment of diverse COVID-19 vaccines, primarily targeted at individuals with a high risk of infection and severe disease [3,4,5].

The expedited vaccine development process predominantly centred on the viral spike protein [6], with notable formulations including mRNA vaccines such as mRNA-1273 (Spikevax, Moderna—NIAID) and BNT162b2 (Comirnaty, Pfizer—BioNTech), as well as the ChAdOx1-S (Vaxzevria, AstraZeneca—University of Oxford) and Ad26.COV2.S (Jcovden, Janssen). The first to gain approval was the BNT162b2 vaccine on 31 December 2020 [4].

The mRNA vaccine mechanism involves isolating the SARS-CoV-2 virus’s mRNA encoding the spike protein, encapsulating it in a lipid nanoparticle, and delivering it intramuscularly. Within host cells, the mRNA is translated by ribosomes to synthesize spike proteins, triggering an immune response. A modified chimpanzee DNA adenovirus serves as the vector for the AstraZeneca vaccine, generating an immune response against the viral protein encoded in the host DNA [7,8,9].

It is crucial to emphasize that the effectiveness of COVID-19 vaccines can vary based on factors, such as viral variants, population demographics, and other considerations. After vaccination, immunity against SARS-CoV-2 in the respiratory mucosa primarily involves humoral immunity, with IgG prevailing over IgA titres against vaccine antigens. Individuals exhibited detectable levels of spike IgG in the airway mucosa, and their level was increased when accompanied by mucosal IgA in infected patients. [10] 

In the intricate interplay between metabolic diseases and the evolving landscape of COVID-19, the emergence of the Delta variant introduces a new layer of intricacy [11].

The Delta variant (B.1.617.2), first identified in December 2020 in India, became the most transmissible variant, with 66% more prevalence over the Alpha variant in England and over 79% in some regions from France. In comparison with the previous variants, it has increased severity, higher viral loads, and implies a longer period of virus detection with the polymerase chain reaction (PCR) [12,13,14,15]. Individuals managing metabolic disorders may find themselves at an elevated risk of experiencing severe outcomes when confronted with the Delta variant [16,17]. This intersection emphasizes the urgent need for tailored strategies and nuanced medical interventions to address the unique challenges posed by COVID-19, particularly in individuals burdened by metabolic health issues. Exploring this intricate relationship not only deepens our understanding of the disease’s multifaceted nature but also underscores the imperative of widespread vaccination efforts to mitigate the impact on vulnerable populations.

This study aims to compare the patients characteristics, including demographic parameters, the smoking status, symptoms on hospital admission, comorbidities and CT lung involvement assessed by visual score, Padua score, oxygen saturation (O_2_ saturation), ventilation requirement, dynamic of arterial blood gas (ABG) parameters, ICU admission rate, and mortality rate in vaccinated and non-vaccinated hospitalized patients with the COVID-19 Delta variant, and to evaluate the impact of vaccination in preventing the severity of infection.

## 2. Materials and Methods

### 2.1. Study Population

This retrospective study, conducted between October 2021 and February 2022, investigated a cohort of 226 adult patients admitted to a COVID-19 department at a University Centre in Cluj-Napoca, Romania. The study adhered to the guidelines of the Declaration of Helsinki, and approval from the Human Research Ethics Committee (number: 60/2023) was obtained.

Inclusion criteria for patients were (1) patients with laboratory-confirmed SARS-CoV-2 infection through PCR test, (2) aged ≥ 18 years, (3) hospitalized in our unit, and (4) vaccinated and unvaccinated patients with Delta variant.

We excluded the patients with (1) insufficient laboratory or imaging records (n = 21 patients) and (2) patients transferred to other units (n = 8 patients).

**Figure 1:**
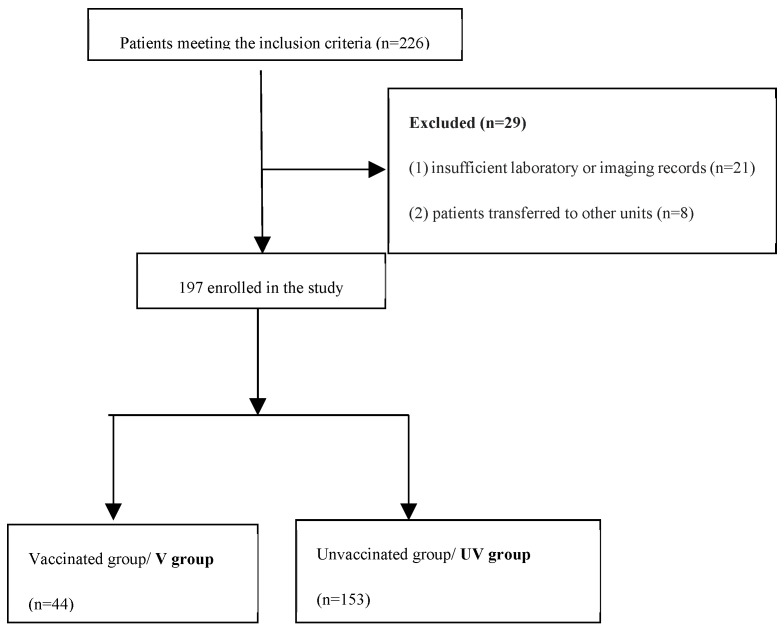
Study flowchart.

Finally, 197 participants were eligible for taking part in the study. They were divided into two groups: 44 patients were fully vaccinated (group V) and 153 were unvaccinated (UV). Enrolment of the patients is described in Figure 1.

The confirmation of COVID-19 was determined through PCR testing conducted on samples obtained from either nasopharyngeal swabs or sputum. Additionally, abnormal findings in computed tomography scans supported the diagnosis. Notably, the genetic analysis revealed that all individuals tested positive for the Delta variant, a significant concern at the time of the SARS-CoV-2 virus spreading. This determination was based on the distinctive genetic markers identified during the PCR testing process, enabling the specific identification of the Delta variant in each confirmed case. The vaccination status and the type of the vaccine was checked on the proof of vaccination. The term “fully vaccinated” refers to an individual who has received the complete dose regimen of COVID-19 vaccine as recommended by the relevant health authorities or vaccine manufacturers at the time (a two-dose regimen of mRNA-1273, BNT162b2, and ChAdOx1-S, and a single-dose regimen of Ad26.COV2.S).

Mild or moderate cases were generally defined based on discomfort and illness that can be managed at home with mild clinical symptoms (low grade fever, cough, discomfort, shortness of breath, or fatigue) and recovery within a couple of weeks with rest, hydration, and over-the-counter medications to alleviate symptoms [18,19].

The severity of the SARS-CoV-2 infection was defined according to the national and international criteria: a lung’s involvement > 50% on CT scan, scored by visual assessment; hypoxia, defined by a drop in oxygen saturation SpO_2_ < 94%; PaO_2_/FiO_2_ < 300 mm Hg; or a respiratory rate (RR) > 30/min.

The Padua score, originally developed for estimating venous thromboembolism (VTE) risk in hospitalized patients, has been used to help identify COVID-19 patients who are at higher risk for VTE and who may benefit from prophylactic anticoagulation therapy. Studies have shown that COVID-19 patients have an increased risk of VTE due to the hypercoagulable state associated with the pro-inflammatory response to the pathogen [20].

Patients were treated according to Romanian National Guidelines, receiving a standard care regimen inclusive of dexamethasone, anticoagulants, antibiotics, nutritional support, hepatic and gastric protective medications, and oxygen therapy. Severe cases received antivirals (remdesivir) and immunomodulators (tocilizumab or anakinra). [21]. 

The administration of oxygen supplementation was initiated in the severe cases of COVID-19 (oxygen saturation ≤ 93% on room air) when patients experienced significant respiratory distress.

### 2.2. Data Collection: Clinical Characteristics and Blood Sample Collection

Medical records provided data on demographic parameters, smoking status, symptoms at hospital admission, comorbidities, CT lung involvement (visual score), Padua score, O_2_ saturation, ventilation requirement, ABG parameters, ICU admission rate, and mortality rate.

Arterial blood samples (ABG) were collected at admission and daily during the hospital stay. An anaerobic 2 to 3 mL of arterial specimen was obtained from a peripheral artery using a 3 or 5 mL airtight syringe. Analysis occurred within 10 min. 

### 2.3. Statistical Analysis 

Descriptive and inferential statistical methods were applied. Factor variables were described using frequency and percentages; continuous variables used mean, median, and interquartile range. Pearson’s Chi-square test and the Wilcoxon rank-sum test were used for nominal and continuous variables. Ordinal regression and univariate/multivariate linear regressions were performed. Significance was considered for *p*-value < 0.05. Analysis used R (version 2022.12.0+353) with RStudio (version 2022.12.0+353 for Mac).

## 3. Results

### 3.1. Patient’s Characteristics, Demographic Parameters, the Smoking Status, Symptoms on Hospital Admission, Comorbidities, Vaccine Type, and the Delay between Complete Vaccination–Infection (Table 1, Table 2, Table 3, Table 4 and Table 5)

We analysed a total of 197 patients, comprising 47 vaccinated and 153 unvaccinated individuals. The groups were homogeneous concerning age, body mass index (BMI), and smoking status. Notably, a statistically significant difference was observed in sex, with more women in the unvaccinated group (*p* = 0.008). Both groups exhibited comparable symptoms on hospital admission and comorbidities.

Table 1 reveals a statistically non-significant difference in age between vaccinated (mean = 59) and unvaccinated (mean = 64) groups (*p* = 0.10). BMI mean values were 28.3 and 29.0 for vaccinated and unvaccinated groups, respectively (*p* = 0.3). The smoking status distribution did not differ significantly between the groups (*p* = 0.14).

Table 3 displays similar symptomatology on hospital admission between vaccinated and unvaccinated groups, with no significant differences observed.

Table 4 demonstrates no significant variations in comorbidities between the groups.

Table 5 shows a longer period of time between Pfizer vaccine and infection, translating to a longer period of immunization.

**Table 1:** Patients’ characteristics.

Characteristic	Vaccinated, n = 44 ^1^	Unvaccinated, n = 153 ^1^	*p*-Value ^2^
**Sex**			0.008
Females	16 (36%)	90 (59%)	
Males	28 (64%)	63 (41%)	
**Age**			0.10
Mean (SD)	59 (16)	64 (15)	
Median (IQR)	62 (50, 72)	66 (54, 74)	
**Body Mass Index**			0.3
Mean (SD)	28.3 (4.8)	29.0 (4.9)	
Median (IQR)	27.7 (25.2, 31.2)	28.0 (26.0, 32.6)	

Abbreviations: ^1^ n (%); ^2^ Pearson’s Chi-squared test; Wilcoxon rank-sum test; IQR, interquartile range; SD, standard deviation.

**Table 2:** Smoking status.

Characteristic	Vaccinated, n = 44 ^1^	Unvaccinated, n = 153 ^1^	*p*-Value ^2^
Smoking status			0.14
Smoker	10 (23%)	41 (27%)	
Ex-smoker	10 (23%)	17 (11%)	
Non-smoker	24 (55%)	95 (62%)	

Abbreviations: ^1^ n (%); ^2^ Pearson’s Chi-squared test; Wilcoxon rank-sum test; IQR, interquartile range; SD, standard deviation.

**Table 3:** Patients’ symptoms on hospital admission.

Symptoms on Hospital Admission	Vaccinated, n = 44 ^1^	Unvaccinated, n = 153 ^1^	*p*-Value ^2^
Fever			0.7
YES	31 (70%)	112 (73%)	
NO	13 (30%)	41 (27%)	
Chills			0.2
YES	32 (73%)	95 (62%)	
NO	12 (27%)	58 (38%)	
Cough			0.6
YES	34 (77%)	113 (74%)	
NO	10 (23%)	40 (26%)	
Dyspnea			0.3
YES	24 (55%)	97 (63%)	
NO	20 (45%)	56 (37%)	
Arthralgia			0.7
YES	6 (14%)	28 (18%)	
NO	38 (86%)	124 (81%)	
Myalgias			0.8
YES	13 (30%)	49 (32%)	
NO	31 (70%)	104 (68%)	
Diarrhea			0.13
YES	1 (2.3%)	16 (10%)	
NO	43 (98%)	137 (90%)	

Abbreviations: ^1^ n (%); ^2^ Pearson’s Chi-squared test; Wilcoxon rank-sum test; IQR, interquartile range; SD, standard deviation.

**Table 4:** Patients’ comorbidities.

Comorbidities	Vaccinated, n = 44 ^1^	Unvaccinated, n = 153 ^1^	*p*-Value ^2^
Obesity			0.3
YES	16 (36%)	68 (44%)	
NO	28 (64%)	85 (56%)	
Diabetes			0.5
YES	15 (34%)	43 (28%)	
NO	29 (66%)	109 (72%)	
Arterial Hypertension			0.8
YES	26 (59%)	94 (61%)	
NO	18 (41%)	59 (39%)	
Cardiovascular disease			0.4
YES	21 (48%)	63 (41%)	
NO	23 (52%)	90 (59%)	
Respiratory disease			0.8
YES	8 (18%)	31 (20%)	
NO	36 (82%)	122 (80%)	
Chronic kidney disease			0.7
YES	2 (4.5%)	12 (7.8%)	
NO	42 (95%)	141 (92%)	
CANCER			0.5
YES	4 (9.1%)	8 (5.2%)	
NO	40 (91%)	145 (95%)	

Abbreviations: ^1^ n (%); ^2^ Pearson’s Chi-squared test; Wilcoxon rank-sum test; IQR, interquartile range; SD, standard deviation.

**Table 5:** Vaccine type and delay between complete vaccination and admission date.

Vaccin Manufacturer	Average Difference between Vaccination and Admission Date (Months)
*BNT162b2*	6
*ChAdOx1-S*	5
*Ad26.COV2.S*	2

### 3.2. Disease Severity Assessed by CT Score and Padua Score 

**Figure 2:**
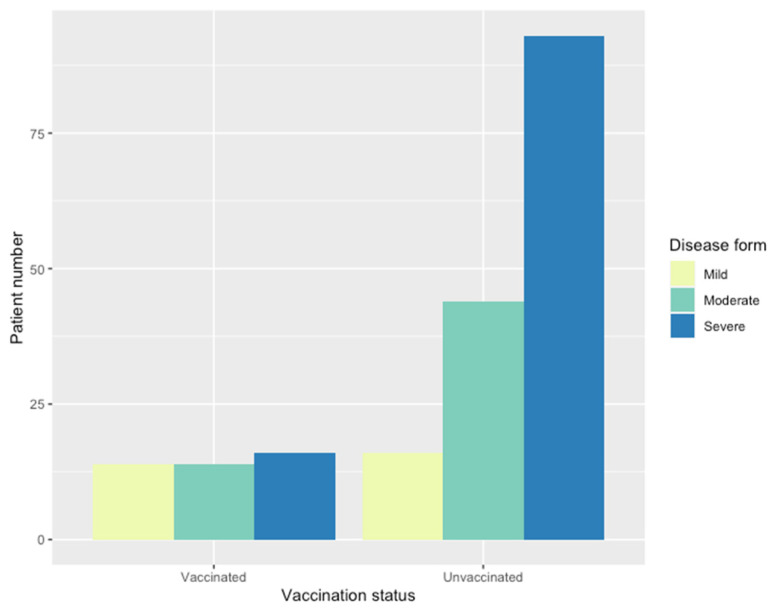
Relative frequency for disease form based on vaccination status.

The severity of COVID-19, assessed by CT visual score and Padua score, revealed significant differences between vaccinated and unvaccinated groups. The unvaccinated group exhibited a higher frequency of severe forms of the disease (60% vs. 36%, *p* < 0.001) (Figure 2).

**Figure 3:**
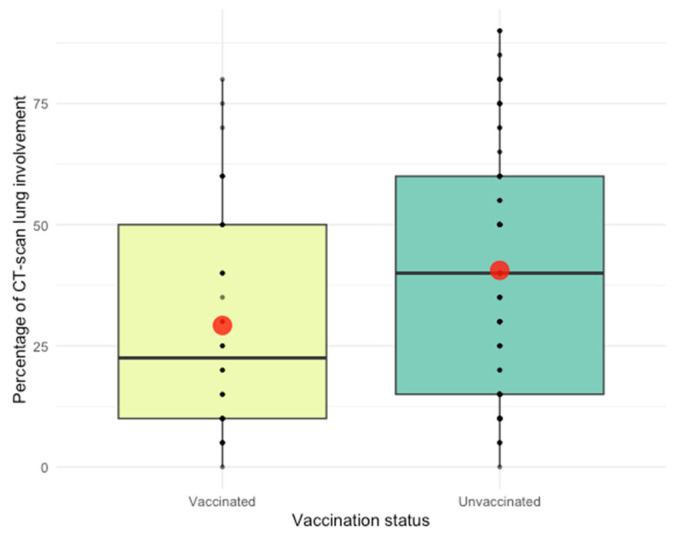
CT scan showing the lung’s involvement distribution in vaccinated and unvaccinated patients. (The middle line of the boxplot represents the median, the upper and lower bars represent the 75th and 25th percentile, and the red dot represents the mean).

An ordinal regression model (Table 6) indicated that unvaccinated individuals had 3.5 times higher odds of a severe disease form. CT scan determined pulmonary involvement was significantly higher in the unvaccinated group (median = 40%) compared to the vaccinated group (median = 22%, *p* = 0.005) (Figure 3).

Multivariate regression analysis (Table 7) highlighted significant associations between lung involvement and vaccination status, with unvaccinated patients showing 11.5% higher lung involvement.

### 3.3. Padua Score 

**Figure 4:**
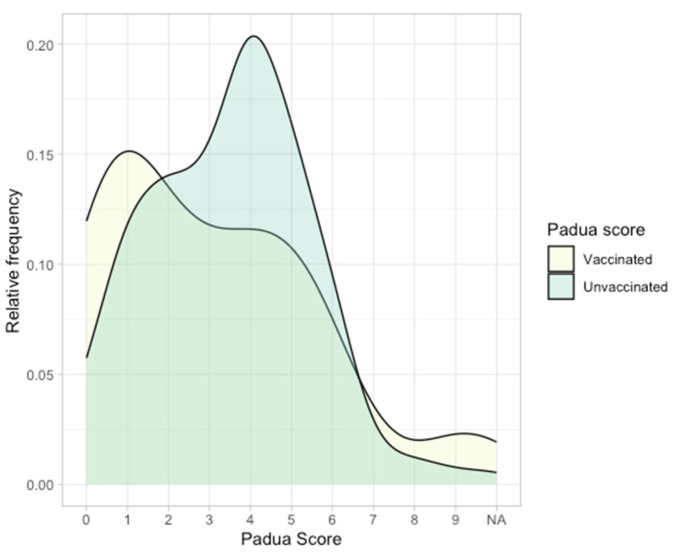
Density plot showing the Padua score distribution in the studied groups.

For the Padua score, median values did not differ significantly (Wilcoxon rank-sum test *p* = 0.07). Distribution levels are displayed in Figure 4.

We have compared two linear regression models using the Padua score, one with and one without vaccination status as a prediction variable. ANOVA analysis was not statistically significant (*p* = 0.38), so we concluded that the Padua score was not influenced by vaccination status. Univariate and multivariate linear regression results of the Padua score, including the vaccination status, age, sex, and BMI, are displayed in Table 8.

### 3.4. Oxygen Therapy, Ventilation Requirement and the Dynamic of ABG 

Oxygen requirement comparisons revealed higher needs in the unvaccinated group at hospital admission and during hospitalization (*p* = 0.003, *p* = 0.047) (Table 9). Non-invasive ventilation was more frequent in the unvaccinated group.

**Figure 5:**
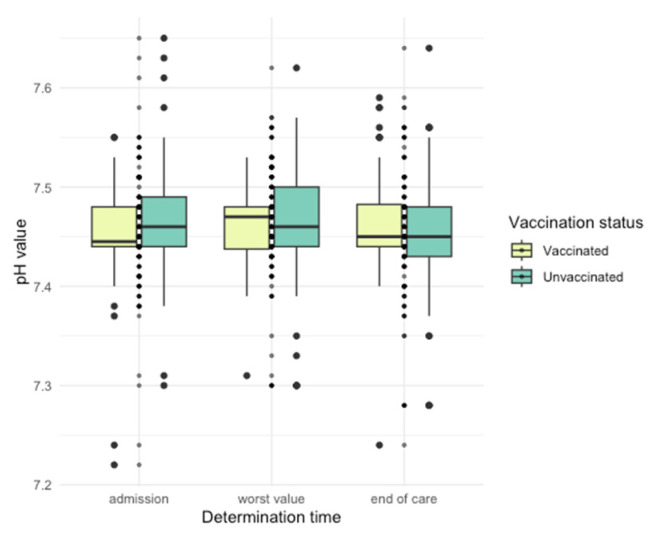
pH values among vaccinated and unvaccinated patients at different time points.

**Figure 6:**
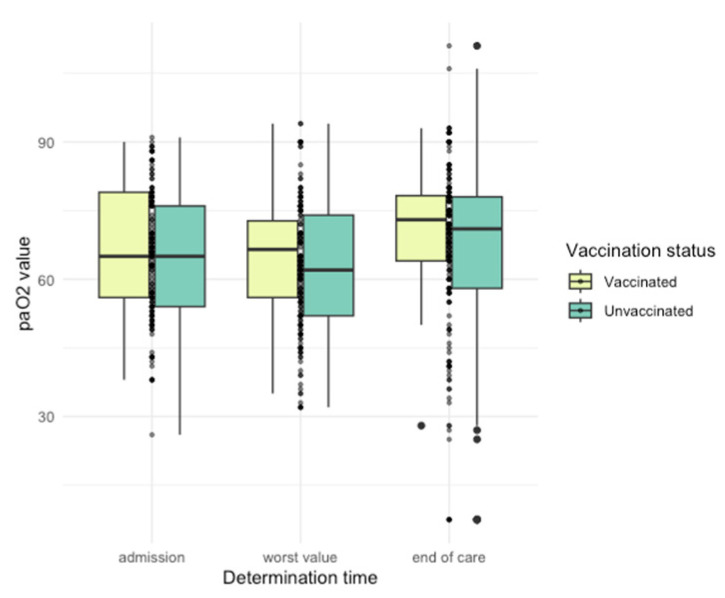
PaO_2_ values among vaccinated and unvaccinated patients at different time points.

**Figure 7:**
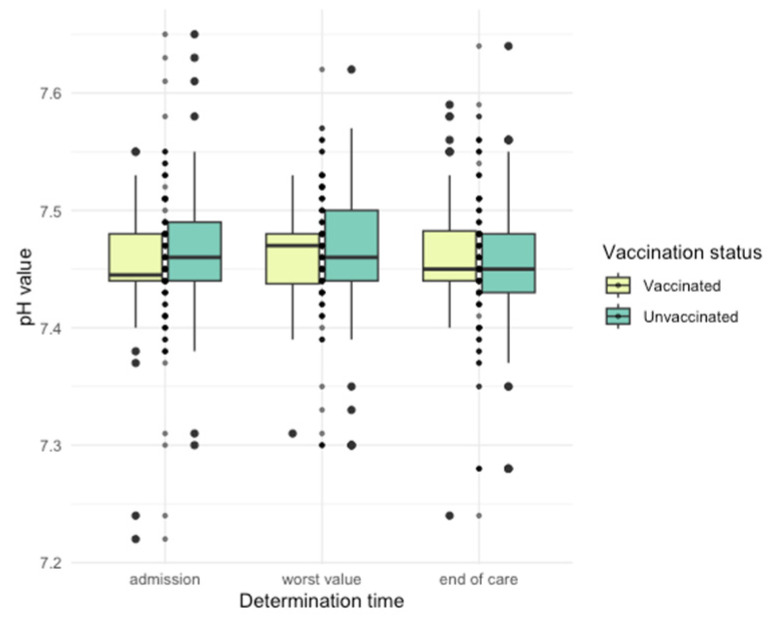
PCO_2_ values among vaccinated and unvaccinated patients at different time points.

**Figure 8:**
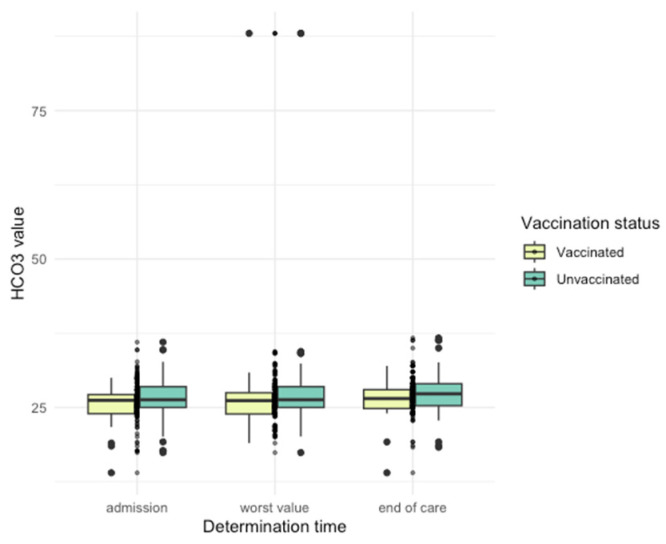
HCO_3_^−^ values among vaccinated and unvaccinated patients at different time points.

**Figure 9:**
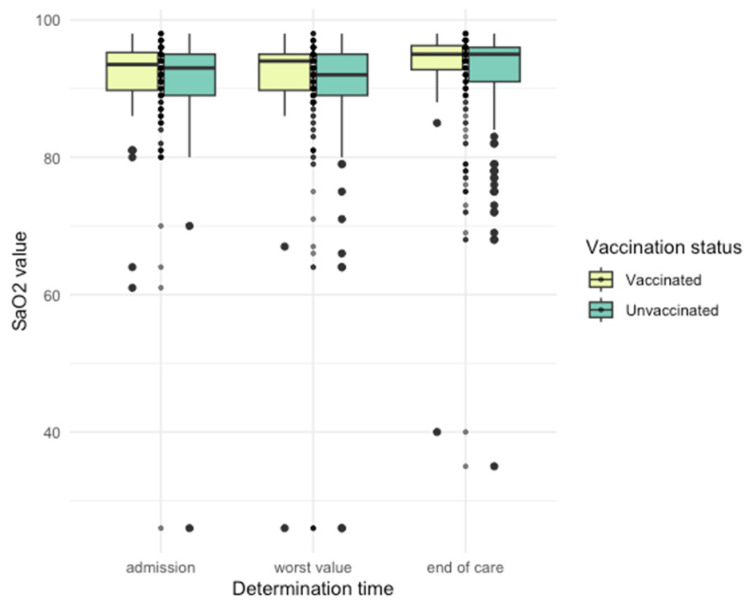
SaO_2_ values among vaccinated and unvaccinated patients at different time points.

Differences in arterial blood gas (ABG) parameters were observed (Figure 5, Figure 6, Figure 7, Figure 8 and Figure 9), with unvaccinated individuals showing higher pCO_2_ values at discharge (*p* < 0.001).

### 3.5. ICU Admission and Mortality

ICU admission and mortality rates were significantly higher in the unvaccinated group (*p* = 0.07, *p* = 0.03) (Table 10). Stratification by disease severity demonstrated a significant association between disease severity and ICU admission/mortality (Table 11).

**Figure 10:**
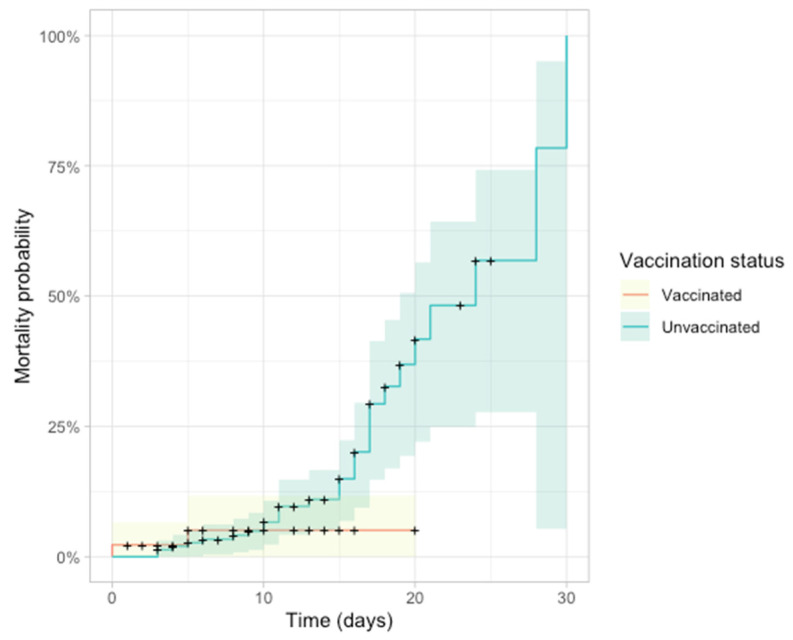
Kaplan–Meier plot of mortality probability based on vaccination status.

Figure 10 illustrates the Kaplan–Meier plot, indicating higher mortality probability in the unvaccinated group after day 20.

## 4. Discussion

The emergence of the Delta variant has introduced unprecedented challenges to our understanding and management of COVID-19 [22,23]. This highly transmissible variant has not only complicated the existing narrative of the pandemic but has also underscored the dynamic nature of the virus, requiring swift adaptations in our approach to diagnosis, treatment, and prevention. The ongoing evolution of the virus necessitates a continuous pursuit of knowledge and the development of flexible strategies to effectively navigate the complexities introduced by emerging variants [24,25]. In this study, we have classified the patients infected with the Delta variant by their vaccination status to further explore the vaccination’s impact on disease severity and outcomes.

Our meticulous focus on patients infected with the Delta variant reveals compelling evidence regarding the efficacy of vaccination against severe disease associated with this highly transmissible strain. While breakthrough infections occurred among both fully vaccinated and unvaccinated individuals, our findings underscore the vaccine’s protective advantage, particularly in preventing severe forms of the illness.

In the context of the Delta variant, our study aligns with and extends existing research on the efficacy of COVID-19 vaccines. Notably, our results contribute to the growing body of evidence supporting the vaccines’ resilience against the Delta variant [26,27].

A preprint study analysing data from Scotland, found that the Pfizer-BioNTech vaccine was 79% effective at preventing symptomatic infection from the Delta variant. The study also found that the vaccine was 90% effective for preventing hospitalization [28].

A study conducted by Bernal et al. from UK on 19109 patients with the Alpha or Delta variant showed that after one dose of the vaccine (BNT162b2 or ChAdOx1 nCoV-19), the effectiveness was lower in the Delta variant group versus Alpha variant group (30.7% versus 48.7%), and these results were almost the same for both types of vaccines. Regarding the two doses of vaccines, the effectiveness was 93.7% for the Alpha variant and 88% for Delta among those vaccinated with Pfizer. In the ChAdOx1 nCoV-19 vaccine group, the effectiveness was 74.5% for those with the Alpha variant and 67.0% for those with the Delta variant [6].

We showed that the vaccination effectiveness drops after a period of 3–6 months depending on the type of vaccine used, with a shorter period in those vaccinated with Ad26.COV2.S. Our results are aligned with those of Nordström et al. whose study concerned patients previously vaccinated with heterologous immunisation that underwent Delta infection [29]. Feikin et al. in a meta-analysis showed that after 6 months following complete vaccination, there was a decline in vaccine effectiveness against severe disease, averaging between 9.5 and 10.0 percentage points [30]. In a cohort from USA, composed of individuals infected with Delta variant, lower effectiveness was noted in those aged 65 years or older and in those who were administered the Ad26.COV2.S vaccine [31].

While COVID-19 vaccines offer numerous benefits, it is essential to consider the associated risks. Serious adverse events (SAEs) accounted for a significant proportion of reported adverse events (AEs), comprising 25.23% of cases in the VigiBase database. Fatalities occurred in 0.40% of total SAEs attributed to the Pfizer-BioNTech vaccine, with data from clinical trials indicating deaths in two recipients (0.01%), both aged over 55. Non-fatal SAEs were reported in 0.6% of cases, with appendicitis (0.04%), acute myocardial infarction (0.02%), and cerebrovascular accident (0.02%) being the most common. Similarly, fatalities were observed in 1.23% of total SAEs linked to the Moderna vaccine, with myocardial infarction (0.03%), cholecystitis (0.02%), and nephrolithiasis (0.02%) among the frequently reported SAEs. Adverse events associated with the AstraZeneca COVID-19 vaccine, as reported by the Medicines & Healthcare products Regulatory Agency (MHRA), were primarily classified as general disorders and administration site conditions, including injection site reactions/pain, fatigue, headache, and nausea [32].

### 4.1. Top of Form 

The recommendations for COVID-19 vaccination have evolved over time, reflecting the dynamic nature of the pandemic and the emergence of new variants. However, with the emergence of subsequent variants like Omicron, which exhibit even more attenuated disease progression, there has been a discernible shift in vaccination strategies. At the time of our investigation, Romania exhibited one of the lowest vaccination rates in Europe. [33,34].

### 4.2. Bottom of Form

**CT Scan and Lung Involvement**. Our analysis of CT scan findings sheds light on the extent of lung involvement, a critical marker of disease severity. These findings resonate with studies investigating the impact of the Delta variant on pulmonary manifestations. For instance, Kumari et al. demonstrated that complete vaccination led to significantly lower mean CT scores (14.18 ± 7.223) compared to unvaccinated individuals (11.1 ± 6.016) [35]. Another study by Jong Eun Lee et al. found a higher number of negative CT scans in fully vaccinated individuals compared to the unvaccinated group (*p* < 0.001) [36].

**Padua Score and VTE Risk**. Incorporating the Padua score to assess the risk of venous thromboembolism (VTE) adds a valuable dimension to our study. While our results show no significant influence of vaccination status on the Padua score, it remains a crucial tool for assessing VTE risk. This finding resonates with Marietta et al.’s study, which found that a Padua score of four or higher was associated with a significantly higher risk of VTE in COVID-19 patients [37].

**Oxygen Requirement and Respiratory Failure**. Our observations regarding oxygen requirements underscore the severity of respiratory failure, particularly in unvaccinated individuals. These findings align with studies suggesting that vaccination significantly reduces the risk of severe disease, hospitalization, and mortality. For example, Balachandran et al. found that vaccines reduced the risk of non-invasive ventilation need by 48% compared to unvaccinated individuals (OR = 0.52, 95% CI = 0.3–0.91) [27].

**Arterial Blood Parameters**. The significance of oxygen saturation levels in assessing disease severity cannot be overstated. Our study contributes to this understanding, highlighting the potential impact of vaccination on maintaining higher oxygen saturation levels. Korishettar et al.’s study, involving 820 patients, showed that unvaccinated patients had significantly lower mean oxygen saturation levels compared to vaccinated patients [38].

**ICU Admission and Mortality.** Our results reveal a significantly higher rate of ICU admission and mortality among the unvaccinated, affirming the pivotal role of vaccination in preventing severe outcomes. This is consistent with global studies, including one conducted by Public Health England, which found that fully vaccinated individuals had an 84% lower risk of hospitalization and an 89% lower risk of death compared to unvaccinated individuals [39].

**Length of Hospitalization**. The relationship between hospitalization length and mortality rates underscores the broader implications of vaccination on patient outcomes. While statistically non-significant, our findings echo studies showing a shorter hospital stay for vaccinated individuals and a potential link between extended hospitalization and increased mortality in unvaccinated patients. Dagan et al.’s study, analysing 1107 COVID-19 patients in Israel, found that the length of hospitalization was significantly shorter for fully vaccinated patients compared to unvaccinated patients [40].

## 5. Limitations

Our study, comparing vaccinated and unvaccinated COVID-19 patients, have some potential limitations. The relatively small number of patients, particularly within the vaccinated group, may introduce a potential source of bias. It is essential to acknowledge that, at the time of our study, the vaccination rate in our country was notably low, reflecting the early stages of vaccine rollout. Another limitation is the selection bias. The inherent risk of selection bias must be acknowledged, as vaccinated individuals might exhibit different health-conscious behaviours or possess distinct risk profiles unrelated to vaccination status. These variations could contribute to differences in outcomes, necessitating cautious interpretation of our findings. Also, the diversity in vaccine types administered to our study population introduces a layer of complexity. Different vaccines may exhibit varying efficacy rates, potential side effects, and levels of protection against the Delta variant. Recognizing this variability is crucial for a comprehensive understanding of our results. Furthermore, timing of vaccination is another bias, because the study included individuals who received vaccines at different times, affecting the level of immunity and susceptibility to the virus. Also, the study’s follow-up period may not be long enough to capture long-term effects of vaccination among the studied population.

## 6. Conclusions

In summary, our study presents compelling insights into the notable efficacy of COVID-19 vaccination against severe SARS-CoV-2 infection, contributing valuable perspectives to the discourse on pandemic management. The observed protective effects of the vaccine are instrumental, demonstrating a significant reduction in the incidence of severe disease, alongside notable decreases in the rates of ICU admission and mortality among hospitalized patients grappling with severe SARS-CoV-2 infection.

Unique to our investigation is the comprehensive examination of oxygen/ventilation requirements and the nuanced analysis of oxygen interface types at various time points. This reveals a distinct pattern of heightened oxygen needs in unvaccinated groups upon hospital admission and during hospitalization. These nuanced findings underscore the pivotal role of vaccination in not only preventing severe disease outcomes but also in shaping the trajectory of oxygen supplementation needs.

As we navigate the complexities of the COVID-19 landscape, our study positions vaccination as a paramount strategy in mitigating hospitalization, ICU admissions, and mortality. The evidence presented herein reinforces the centrality of vaccination as a frontline medical intervention, establishing its indisputable efficacy in combatting the challenges posed by the COVID-19 pandemic. Moving forward, our findings contribute to the evolving understanding of vaccination outcomes, emphasizing its pivotal role in public health strategies aimed at reducing the burden of severe SARS-CoV-2 infections.

## Figures and Tables

**Table 6:** Ordinal regression table for the form of disease.

Prediction Variable (Compared Variable)	Odds Ratio	CI: 2.5–97.5%	*p*-Value
**Vaccination status (Unvaccinated)**	3.50	1.71–7.31	<0.001
**Sex (Males)**	2.38	1.27–4.57	<0.001
**Age**	1.04	1.02–1.06	<0.001
**BMI**	1.16	1.08–1.24	<0.001
**Smoking status (Smoking)**	0.97	0.47–2.02	0.93
**Smoking status ** **(Ex-smoker)**	1.53	0.58–4.30	0.4

Abbreviations: CI, confidence interval. Reference categories are females for sex, vaccinated for vaccination status, and non-smoker for smoking status.

**Table 7:** Uni and multi-variate regression analysis results for CT scan of lung’s involvement and vaccination status.

	Univariate Analysis			Multivariate Analysis		
Characteristic	Beta	95%CI	*p*-Value	Beta	95% CI	*p*-Value
**Vaccination status**						
**Vaccinated**	reference	—		reference	—	
**Unvaccinated**	11	3.2, 20	0.006	**11.5**	3.6, 19	0.004
**Sex (Male/Female)**						
**Female**	reference	—		reference	—	
**Male**	5.5	−1.4, 12	0.12	6.7	−0.17, 14	0.056
**Age (years)**	0.15	−0.08, 0.38	0.2	0.15	−0.08, 0.38	0.2
**BMI**	1.7	1.1, 2.4	<0.001	1.6	0.93, 2.3	<0.001
**Smoking status**						
**Not smoking**	reference	—		reference	—	
**Smoker**	5.1	−3.0, 13	0.2	1.5	−6.5, 9.4	0.7
**Ex-smoker**	9.2	−1.0, 20	0.077	4.7	−5.4, 15	0.4

Abbreviations: CI, confidence interval.

**Table 8:** Linear regression results for the Padua Score.

	Univariate Analysis			Multivariate Analysis		
Characteristic	Beta	95% CI	*p*-Value	Beta	95% CI	*p*-Value
**Vaccination status**						
**Vaccinated**	Reference			Reference	—	
**Unvaccinated**	0.55	−0.14, 1.2	0.12	0.27	−0.35, 0.90	0.4
**Sex**						
**Female**	Reference			Reference	—	
**Male**	−0.03	−0.60, 0.54	>0.9	0.23	−0.29, 0.75	0.4
**Age (years)**	0.05	0.04, 0.07	<0.001	0.05	0.04, 0.07	<0.001
**BMI**	0.12	0.06, 0.17	<0.001	0.11	0.06, 0.17	<0.001

Abbreviations: BMI, body mass index; CI, confidence interval.

**Table 9:** Oxygen therapy and ventilation requirements based on vaccination status.

Characteristic	Vaccinated, n= 44 ^1^	Unvaccinated, n = 153 ^1^	*p*-Value ^2^
Need for oxygen support on admission time			**0.003**
Ambiental air	17 (39%)	21 (14%)	
Nasal canula	13 (30%)	77 (50%)	
Reservoir mask	13 (30%)	40 (26%)	
Simple facial mask	1 (2.3%)	12 (7.8%)	
Venturi mask	0 (0%)	3 (2.0%)	
Need for oxygen support: worst			**0.047**
Ambiental air	16 (36%)	21 (14%)	
Nasal canula	9 (20%)	47 (31%)	
High Flow Nasal Cannula	0 (0%)	9 (6%)	
Reservoir mask	14 (32%)	59 (39%)	
Simple facial mask	4 (9.1%)	11 (7.2%)	
Non-invasive ventilation	0 (0%)	1 (0.7%)	
Venturi mask	1 (2.3%)	4 (2.6%)	
Need for oxygen support on discharge			0.3
Ambiental air	23 (52%)	58 (38%)	
Nasal canula	11 (25%)	44 (29.7%)	
High Flow Nasal Cannula	0 (0%)	13 (8.5%)	
Reservoir mask	10 (23%)	27 (18%)	
Simple facial mask	0 (0%)	8 (5.2%)	
Non-invasive ventilation	0 (0%)	1 (0.7%)	
Venturi mask	0 (0%)	1 (0.7%)	

Abbreviations: ^1^, number of patients (% of column group); ^2^, Fishers’s exact test.

**Table 10:** Between-group analysis of death and ICU transfer events based on vaccination status.

Characteristic	Vaccinated, n = 44 ^1^	Unvaccinated, n = 153 ^1^	*p*-Value ^2^
**ICU transfer**			0.07
Yes	2 (4.7%)	23 (15%)	
No	41 (95%)	129 (85%)	
**Death**			0.030
Yes	2 (4.5%)	27 (18%)	
No	42 (95%)	125 (82%)	

^1^, n(%); ^2^, Pearson’s Chi-squared test; Fisher’s exact test.

**Table 11:** Between-group analysis of death and ICU transfer events based on disease severity.

Characteristic	Mild, n = 30 ^1^	Moderate, n = 58 ^1^	Severe, n = 109 ^1^	*p*-Value ^2^
**ICU transfer**				<0.001
Yes	1 (3.3%)	1 (1.7%)	23 (21%)	
No	29 (97%)	57 (98%)	84 (79%)	
**Death**				<0.001
Yes	0 (0%)	0 (0%)	29 (27%)	
No	30 (100%)	58 (100%)	79 (73%)	

^1^, n(%); ^2^, Pearson’s Chi-squared test; Fisher’s exact test.

## Data Availability

The data presented in this study are available on request from the first author.
